# Geo-Social Top-*k* and Skyline Keyword Queries on Road Networks

**DOI:** 10.3390/s20030798

**Published:** 2020-02-01

**Authors:** Muhammad Attique, Muhammad Afzal, Farman Ali, Irfan Mehmood, Muhammad Fazal Ijaz, Hyung-Ju Cho

**Affiliations:** 1 Department of Software, Sejong University, Seoul 05006, Korea; mafzal@sejong.ac.kr (M.A.); farmankanju@sejong.ac.kr (F.A.); 2 Faculty of Engineering & Informatics, University of Bradford, Bradford BD7 1DP, UK; irfanmehmood@ieee.org; 3 Department of Industrial and Systems Engineering, Dongguk University, Seoul 04620, Korea; fazal@dongguk.edu; 4 Department of Software, Kyungpook National University, Sangju-Si 37224, Korea

**Keywords:** top-*k* spatial keyword queries, skyline queries, location-based social networks, geo-social queries

## Abstract

The rapid growth of GPS-enabled mobile devices has popularized many location-based applications. Spatial keyword search which finds objects of interest by considering both spatial locations and textual descriptions has become very useful in these applications. The recent integration of social data with spatial keyword search opens a new service horizon for users. Few previous studies have proposed methods to combine spatial keyword queries with social data in Euclidean space. However, most real-world applications constrain the distance between query location and data objects by a road network, where distance between two points is defined by the shortest connecting path. This paper proposes geo-social top-*k* keyword queries and geo-social skyline keyword queries on road networks. Both queries enrich traditional spatial keyword query semantics by incorporating social relevance component. We formalize the proposed query types and appropriate indexing frameworks and algorithms to efficiently process them. The effectiveness and efficiency of the proposed approaches are evaluated using real datasets.

## 1. Introduction

Smartphones and social networks are significant innovations from the past decade, and the combination of these technologies has engendered geo-social network (GSN) applications, such as Facebook, Instagram, and Foursquare. Geo-tagged data (e.g., photos, videos, check-ins, and likes) allow these applications to provide many useful services to users based on social and location relevance. Furthermore, the easy availability of textual descriptions for desired facilities (e.g., restaurants, departmental stores, and travel destinations) has promoted many decision support systems and recommendation services.

Traditional top-*k* spatial keyword queries [1,2,3] rank facilities based on spatial proximity to the query location and textual relevance to query keywords. Many existing studies have proposed spatial keyword query systems in Euclidean space [3,4,5] and road networks [6,7]. However, query results can be improved by including social data, since users tend to consult other users and specially their friends, besides their own preferences, for recommendations on movies, restaurants, and places to visit. Therefore, this paper investigates geo-social keyword queries that not only exploit spatial and textual information, but also social information to offer many interesting services. Each data object has a set of fans who exhibit positive behavior towards it, and social relevance is obtained from the number of fans and the relationship between these fans and the query user.

Geo-social keyword queries can be used for a wide range of GSN applications and services, such as a tourist visiting Seoul and searching for a French restaurant. Geo-social keyword queries uses spatial, textual, and social information parameters to retrieve the query results. Spatial information relates to the distance between the user and the facility, textual relevance relates to how well the facility description matches query keywords, e.g., French restaurant; and social information relates to a tourist’s friends and other users.

These query types are also important in various monitoring systems, such as disease monitoring and crime prevention (e.g., users searching about drugs and subsequently joining Facebook pages to discuss drug related activities). Law enforcement agencies can identify crime locations by monitoring commonly visited places for users of those pages. Similarly, Dengue fever patients can be connected using social networks, and the monitoring of frequently visited places could help medical teams identify key disease spreading locations. The queries can also be used for decision support systems such as determining the best location to open a new business or store.

Geo-social queries [8,9,10] have recently attracted significant research attention due to their real-world relevance. Sohail et al. [9] proposed a method to monitor socio-spatial top-*k* famous queries and skyline queries in Euclidean space. However, they did not consider the textual relevance to the query keyword. Wu et al. [10] investigated top-*k* social aware keyword queries in Euclidean space. However, users typically follow the road network to reach their desired location. Moreover, processing spatial queries on road networks is significantly more complicated than in Euclidean space because, it requires computing several shortest paths. Therefore, Euclidean space algorithms cannot be applied to road networks.

Therefore, we propose geo-social top-*k* keyword (GSTK) queries to retrieve the *k* best data objects based on spatial, textual and social relevance. However, the results depend on the scoring function defined by the user and choosing a suitable scoring function may be challenging due to different attribute distributions or inadequate user knowledge. Hence, we also introduce geo-social skyline keyword (GSSK) queries, which do not require scoring. GSSK queries return every object for which there does not exist any other object that has a higher spatial, textual, and social score. In this study, we formalize the concept of processing these queries and provide methodology to process and rank objects considering spatial, textual, and social relevance. We provide a formal definition of GSTK and GSSK in Section 4.1 and Section 5.1, respectively.

The main contributions of this study are summarized as follows:

- We introduce geo-social top-*k* keyword (GSTK) queries on road networks that ranks data objects based on spatial, textual and social relevance.
- We extend our work to propose geo-social skyline keyword (GSSK) queries that return data objects that are not dominated by any other data object.
- We present an indexing technique to retrieve data objects. Furthermore, we present efficient algorithms that exploit the indexing technique to process these queries.
- Finally, we conduct extensive experiments on real road network datasets to demonstrate the efficiency of the proposed techniques.

## 2. Related Work

In this section, we discuss previous studies related to our work. Section 2.1 briefly reviews top-*k* keyword queries. Section 2.2 discusses skyline queries. Finally, Section 2.3 presents a survey on geo-social queries.

### 2.1. Top-*k* Keyword Queries

Several approaches have been proposed to rank spatial data objects based on keyword relevance. Initially, Zhou et al. [11] proposed hybrid indexing methods that combine inverted indexes [12] for text processing and R*-tree [13] for spatial processing. Cong et al. [4] and Li et al. [5] introduced top-*k* spatial keyword queries where each object is ranked based on its combined textual and spatial relevance to query keywords and location. Both studies generated IR-trees by integrating spatial indexing and text indexing. In contrast to Zhou et al. [11] who applied text indexes to filter web documents and then used spatial indexes to process location, the IR-tree [4,5] combines indexes to prune the search space. Rocha et al. [14] proposed an S21 indexing structure to map each keyword to a block or aggregated R-tree for frequent terms.

Recent studies have investigated several spatial queries, such as nearest neighbor, reverse nearest neighbor, range, and various top-*k* queries for road networks [15,16,17,18,19]. Rocha et al. [7] considered top-*k* spatial keyword queries for road networks, and proposed an efficient indexing technique and an overlay network to group objects in regions with similar textual description, thereby enabling the computation of upper-bound scores for all objects in the region. Gao et al. [20] presented filter-and-refinement based algorithms to process reverse top-*k* boolean spatial keyword queries on road networks. Guo et al. [6] investigated the problem of continuous top-*k* spatial keyword queries on road networks and proposed two algorithms to monitor continuous top-*k* keyword queries incrementally that minimize the expansion of the network edges. Attique et al. [21] recently expanded the problem and proposed a safe region based approach to monitor moving top-*k* keyword queries in directed and dynamic road networks, where each network edge is directed and its traveling cost depends on the traffic conditions.

### 2.2. Skyline Queries

Borzsony et al. [22] studied skyline queries and proposed two approaches: block nested loop (BNL) and divide and conquer (D&C). Subsequently, Chomicki et al. proposed a sort filter skyline SFS [23] to reduce skyline evaluation cost by sorting the objects before applying BNL. The sorting technique improves performance because objects can only dominate the subsequent objects. Sharifzadeh et al. [24] introduced spatial skyline queries, which are useful for many location-based applications, decision-support systems and recommendation systems. Deng et al. [25] proposed an algorithm to process multi-source skyline queries in road networks [26], defined a keyword matched skyline query to obtain the set of objects whose textual description contain all query keywords. However, they did not consider a distance function to obtain skyline objects.

Regaldo et al. [27] and Shi et al. [28] recently investigated location-based textual skyline (LTS) and spatio-textual skyline (STS) queries, respectively. Both queries retrieve objects of interest based on Euclidean distance to query location and textual relevance to a set of query keywords. Two algorithms were proposed in [27], but main limitation of their work was that they only considered a single query location. Shi et al. [28] proposed three models to integrate textual relevance into the spatial skyline, with spatio-textual dominance (STD) being the most efficient, because it integrates spatial distance and textual relevance to effectively prune irrelevant objects. Both studies considered textual and Euclidean distance functions to find skyline objects. However, this study considers geo-social keyword skyline queries that return the dominant objects based on their aggregated score of social relevance to the query, textual similarity to the query keywords, and network distance to the query location through the shortest path. Therefore, their objectives and problem formulations are entirely different and cannot be applied to process GSSK queries for road networks.

### 2.3. Geo-Social Queries

Geo-Social query processing is an emerging field that has recently garnered considerable attention from the research community [29,30,31,32,33]. Huang et al. [34] proposed geo-social network services that organize users in social networks based on geographic features, retrieving the set of nearby users that share common interests. Ye et al. [35], designed a location recommendation system based on a user’s social networks. Sarwat et al. [36] also proposed a location-aware recommendation system that associates a user’s social network(s) and locations with ratings. Liu et al. [37] proposed a circle of friends query that returns a group of friends in the user’s geo-social network who are geographically and socially close to each other (e.g., community services, friend gathering, and combined sports activities). Shim et al. recently studied the *k*-nearest *l*-close friends query, which reports the *k* nearest data objects to a query location based on *l*-hop friends in the social network. Zhao et al. [31] proposed a reverse top-*k* keyword query on road networks that finds potential customers for businesses based on spatial, textual, and social information of users.

Emrich et al. [8] introduced geo-social skyline queries that report the set of persons close to a given location, *P* and closely connected to user *U*. Sohail et al. [9] recently introduced top-*k* famous places and socio-spatial skyline queries that consider social and spatial relevance to the query. They provided three approaches: social first, spatial first, and hybrid. The main difference between the GSSK query proposed in the current paper, and the problem studied by Emrich et al. [8] and Sohail et al. [9] is that the previous studies did not consider keyword relevance and their approaches were applied for Euclidean space. Wu et al. [10] investigated a problem similar to the proposed GSTK query. They considered social-aware top-*k* spatial keyword queries, which retrieve objects based on social, spatial, and textual relevance to the query, extended the IR-tree approach, and integrated the social aspect to propose social network aware IR-trees (SNIR-trees). Their approach is also applicable to Euclidean space and relied on R-trees to determine the minimum distance from query location and objects. Algorithms designed for Euclidean space, are not suitable for processing GSTK queries in road networks because they are designed to reduce the number of data objects to be accessed, without considering the underlying spatial network. However, road network based methods should be optimized to minimize the number of network edges to be explored and the cost of computing the distance between query location and objects [25]. To the best of our knowledge, this is the first study to introduce GSTK and GSSK queries in road networks.

## 3. Preliminaries

**Figure 1:**
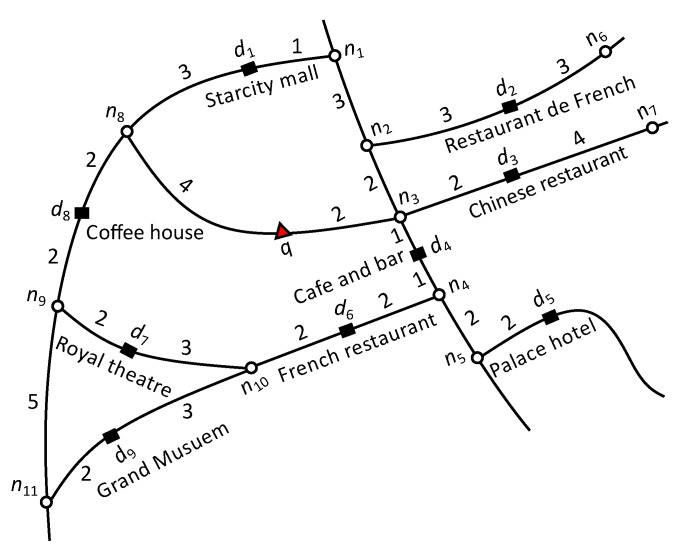
Illustration of road network.

**Road Network:** We represent a road network by an undirected graph G=(N,E,W), where *N* is the set of nodes, *E* is the set of edges, and $W:E\to R^{+}$ is associated with each edge, i.e., a positive real number representing the network weight, such as the distance or travel time. Each edge is represented by a starting and ending node $\left(n_{s},n_{e}\right)$ commonly referred to as boundary nodes $n_{B}\in \left\{n_{s},n_{e}\right\}$. Figure 1 presents a road network example with eleven nodes, $n_{1}$ to $n_{11}$. The query point is represented by a triangle and data objects with their textual description are represented by rectangles. The number on each edge denotes the weight of an edge such as distance in kilometers or travel time. The distance function, $dist(a_{1},a_{2})$ indicates the shortest network distance from $a_{1}$ to $a_{2}$. For example in Figure 1, the shortest distance from *q* to $d_{1}$ is $dist(q,d_{1})=7$ and the shortest path is $q\to n_{8}\to d_{1}$. The set of data objects in an edge $\left(n_{s},n_{e}\right)$ and $\left(n_{e},n_{s}\right)$ are the same, and the $dist(n_{s},d_{i})$ is equal to $dist\left(n_{s},n_{e}\right)-dist\left(n_{e},d_{i}\right)$. Hence, $dist(n_{e},d_{i})$ can be easily obtained from $dist(n_{s},d_{i})$. Therefore, the starting node ($n_{s}$) is used to compute the distance to the objects.

**Figure 2:**
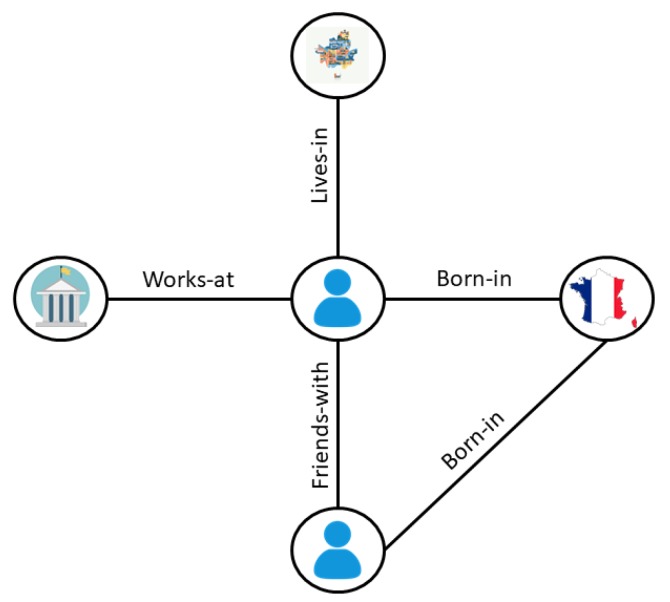
Illustration of geo-social network.

**Geo-Social Networks:** GSNs consist of entities (such as users, groups, and places) and relationships between them. The relationship between two entities *u* and *v* can have several types, such as *friends-with*, *lives-in*, *born-in* and *works-at*. Consider Bob, a Facebook user born in France, who lives in Seoul, works at Sejong University, and is friends with another French user, Alice. Facebook stores this information by connecting Bob and Sejong University with *works-at*, Bob and Alice with *friends-with* and Bob and France with *born-in* relationships. Figure 2 illustrates the resulting social relationship diagram.

**Points of interest:** In this study, points of interest (POIs) are all data objects d∈D such as restaurants, hotels, theme parks, and museums. Each data object lies on the edge *E* of the road network *G*. Each data object has a spatial location d.l, textual description d.t, and set of fans $F_{d}$, where a fan is a user u∈U who exhibits positive behavior towards object *d* (e.g., check-in, like, share, etc.). Table 1 describes the notations used in this study.

## 4. Geo-Social Top-*k* Keyword Queries

### 4.1. Problem Formulation

A geo-social top-*k* keyword (GSTK) query in road network *G* is defined as $Q_{G}=\left(q.l,q.t,q.s,k\right)$, where q.l is the query location, q.t are query keywords, q.s is the query user’s social network, and *k* is the number of desired data objects. Given a set of data objects *D* on *G*; $Q_{G}$ returns *k* data objects from *D* in descending order of score ψ(d), which is defined as:

(1) $$\psi \left(d\right)=\frac{\left[1+\alpha \cdot \mu (q.t,d.t)]\times [1+\beta \cdot \tau (q.s,d.s)\right]}{1+\gamma \cdot \lambda (q.l,d.l)}$$

where μ(q.t,d.t) is the textual similarity between q.t and d.t, τ(q.s,d.s) is the social relevance of *d* with respect to *q*, and λ(q.l,d.l) is the network distance between q.l and d.l. We also defined preference parameters (α,β,γ)∈[0,1] to represent the importance of textual, social and spatial relevance, respectively, with 0 representing the lowest and 1 the highest preference.

Textual relevance (μ) can be measured using any information retrieval model. This study used cosine similarity to compute the relevance between q.t and d.t, which is defined as:

(2) $$\mu \left(q.t,d.t\right)=\sum_{t\in q.t}\Omega_{t(d.t)}.\Omega_{t(q.t)}$$

where the significance $\Omega_{t(n)}=\frac{w_{t(n)}}{\sqrt{\sum_{t\in n}{\left(w_{t(n)}\right)}^{2}}}$ is the normalized weight of the term *t* in the document by considering document length [38,39].

Social relevance (τ) can be expressed as:

$$\tau \left(q.s,d.s\right)=\delta \times \frac{|F_{d}\cap U|}{\left|U\right|}+\left(1-\delta \right)\frac{|N_{q}\cap F_{d}|}{|N_{q}|}$$

where $F_{d}$ represents the set of fans with positive attitude towards data object d∈D (i.e., visited, liked, recommended or shared) and $N_{q}$ represents the adjacent neighbors of user *q*, e.g., if the relationship is works-at and the query entity is the Facebook page “Sejong University,” then $N_{q}$ is a set of all users working at Sejong University. Although any type of relationship can be supported by the presented work, the remainder of this paper only considers friendship relationships for simplicity. In this context, $N_{q}$ comprises only the set of query user’s friends. The expression $\frac{|F_{d}\cap U|}{\left|U\right|}$ represents the portion of users $F_{d}\in U$ who are fans of the data object *d* and $\frac{|N_{q}\cap F_{d}|}{|N_{q}|}$ represents the portion of *q*’s friends who are also fans of *d*. In real life, users commonly consider feedback from friends and other users in social media. Consider a tourist traveling to a foreign country. Typically the farther from home, the more difficult to obtain meaningful opinions from family and friends. Hence, the tourist must rely more on recommendations from other social network users. The proposed social relevance score considers these situations and aggregates the scores $\frac{|F_{d}\cap U|}{\left|U\right|}$ and $\frac{|N_{q}\cap F_{d}|}{|N_{q}|}$. We provide a preference parameter δ to control the importance of one measure over the other, e.g., δ=0 if only friends feedback is considered, δ>0.5 increases the weight of other users feedback over friends feedback, and δ=0.5 meaning equal weight to both sources.

Spatial relevance (λ) is defined as λ(q.l,d.l)=dist(q.l,d.l) which represents the shortest distance between data objects *d* and *q*. Thus, a data object closer to the query has a higher spatial relevance score.

Consider the road network example of Figure 1 and assume that we have a set of users $U=\{u_{1},u_{2},\ldots ,u_{100}\}$. User $u_{1}$ is the query user *q* and issues a query with keywords “French restaurant,” requesting one result (k=1). User $u_{1}$ and has ten friends, $N_{q}=\left\{u_{4},u_{16},u_{23},u_{39},u_{48},u_{55},u_{67},u_{71},u_{80},u_{94}\right\}$. Equal preference is assigned to every attribute, i.e., α=β=γ=1 and δ=0.5. If we only consider spatial relevance, the top-1 result is the nearest restaurant $d_{3}$. If we consider spatial and keyword relevance the top-1 result is $d_{6}$. However, $d_{6}$ does not have a suitable social score. Therefore, $d_{2}$ is the top-1 result considering spatial, textual and social relevance. Notice that although $d_{2}$ is slightly far from $d_{6}$ ($dist\left(q,d_{2}\right)>dist\left(q,d_{6}\right)$) and has less textual relevance than $d_{6}$ ($\mu \left(q.t,d_{2}.t\right)<\mu \left(q.t,d_{6}.t\right)$).

Table 2 summarizes $d_{2}$, $d_{3}$, and $d_{6}$ scores. To simplify the presentation, we assume the textual score is the number of occurrences of the query keywords in the object description divided by the total number of keywords in the object description. For example the textual score of $d_{2}$ is $\frac{2}{3}=0.66$, the social score is $0.5\times \frac{20}{100}+0.5\times \frac{5}{10}=0.35$, and $dist(q,d_{2})=7$. The overall score of $d_{2}$ is computed as $\psi \left(d_{2}\right)=\frac{0.66\times 0.5}{7}=0.033$. Similarly, $\psi (d_{3})=0.02$ and $\psi (d_{6})=0.024$. Consequently, the GSTK query returned $d_{2}$ as the top-1 result.

### 4.2. Indexing Framework

**Figure 3:**
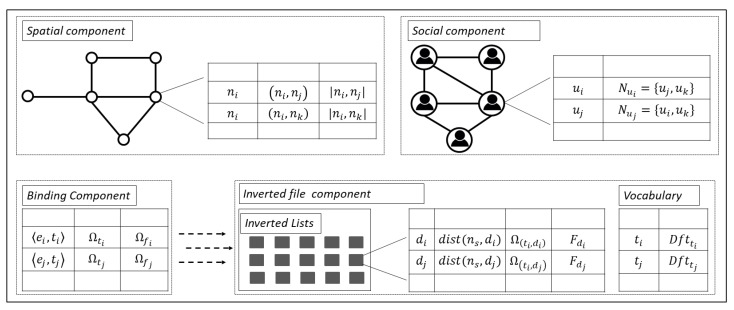
Overview of the proposed indexing framework.

Figure 3 shows the proposed indexing framework structure. The spatial component is used to retrieve adjacent nodes for a given node to allow efficient traversing of the road network. The binding component binds the road network and keywords to the objects using edge id $\left(e_{id}\right)$ and term id $\left(t_{id}\right)$. The social component stores the social relationships of users. Finally, the inverted file component stores data objects along with their descriptions and set of fans.

**Spatial Component:** This component integrates the road network and spatial information as proposed by Papadias et al. [40]. Each road segment is represented by an edge comprising a detailed polyline and is stored in the network’s R-tree. The B-tree is used to retrieve the adjacent nodes of a given node $n_{i}$ from adjacency file, to ensure efficient traversing of the network from one node to the other. The adjacency file stores edge $e_{id}$ (i.e., $\left(n_{i},n_{j}\right)$) along with its weight *W* (i.e., $dist(n_{i},n_{j})$). Data objects that lie on a particular edge can be easily retrieved using $e_{id}$.

**Social Component:** The social component employs a B-tree to index each user $u_{i}\in U$ along with their social relationships $N_{u_{i}}$. The B-tree points to the block in the users file where the social relationships of user are stored. This component enables efficient retrieval of the query user’s relationships to compute the social score.

**Binding Component:** The binding component uses a B-tree that binds a key composed of the pair $e_{id}$ and $t_{id}$ to the inverted lists that contain the data objects located on the edge with term *t* in the data object description. The binding component is also used to efficiently retrieve candidate objects that are relevant to the query based on spatial, textual and social relevance. To achieve this, for each edge the binding component stores the highest significance of a given term *t* ($\Omega_{t}$) among the description of objects lying on the edge and the highest significance of fans ($\Omega_{f}$) of any object lying on the edge with term *t*. The highest significance of a term *t* is an upper-bound textual relevance and the highest significance of fans is the upper-bound social relevance of any object on the edge with *t* in its description. The upper-bound score for edge $e_{id}$ is derived from ($\Omega_{t}$), ($\Omega_{f}$) and the minimum distance from query point to edge. The closest boundary node $n_{B}$ is considered to compute the minimum distance from *q* to $e_{id}$ (i.e., $dist(q,n_{B})$). Therefore, a term *t*’s inverted list on edge $e_{id}$ is accessed only if the upper-bound score is greater than the score of the *k*th object found so far.

**Figure 4:**
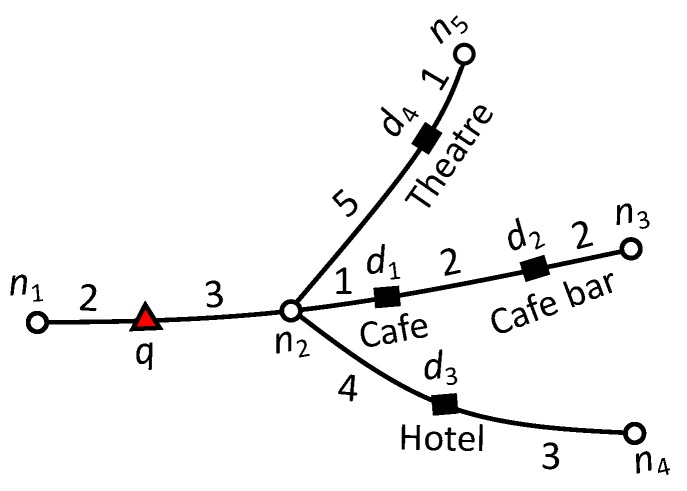
Upper bound score computation.

Consider the example road network presented in Figure 4. We calculate the upper-bound score as follows. Let the set of users be $U=\{u_{1},u_{2},\ldots ,u_{100}\}$, where user $u_{1}$ is the query user who issued a query with the keyword “cafe” and query parameters α=β=γ=1 and δ=0.5. The number of fans of $d_{1},d_{2},d_{3}$ and $d_{4}$ are 15, 30, 40 and 0, respectively. On edge $\left(n_{2},n_{3}\right)$, $\Omega_{t}=1$ since $d_{1}.t=“Cafe”$. To compute the highest significance of social relevance ($\Omega_{f}$), we set the highest score for the portion of friends of *q* who are also fans of *d* i.e., $\frac{|N_{q}\cap F_{d}|}{|N_{q}|}=1$. Thus, for edge $\left(n_{2},n_{3}\right)$ the ($\Omega_{f}=0.5\times \frac{30}{100}+0.5\times 1=0.65$). The minimum distance from *q* to $\left(n_{2},n_{3}\right)$ is $dist(q,n_{2})=3$. Finally, the upper-bound score of edge $\left(n_{2},n_{3}\right)$ is computed as $\frac{1\times 0.65}{3}=0.21$. Similarly, the upper-bound score for edge $\left(n_{2},n_{4}\right)$ is 0 because no data object lie on the edge that contains terms relevant to the query keywords. Finally, the upper-bound score of edge $\left(n_{2},n_{5}\right)$ is 0 because data object $d_{4}$ has no fans and therefore $\Omega_{f}=0.5\times \frac{0}{100}+0.5\times 0=0$.

**Inverted File Component:** This component consists of vocabulary and inverted lists. The vocabulary stores the frequency of each term which assists in computing the textual score of the data objects. The inverted list stores the data objects lying on an edge $e_{i}$ with term *t* in their description. Thus an inverted list stores the distance between the data object and starting node $n_{s}$ ($dist(n_{s},d_{i})$) for edge $e_{id}$, the significance of the term $t_{i}$ in the description of data object ($\Omega_{t_{i},d_{i}}$), and the fans of data object ($F_{d_{i}}$) for each data object $d_{i}$. The social component and fans of data objects stored in the inverted lists are used to compute the aggregated social relevance score. $F_{d_{i}}$ returns all fans of data object $d_{i}$, and the friend list of query $N_{q}$ allows all friends in $F_{d_{i}}$ to be identified. Inverted lists are identified using a ($e_{id},t_{id}$) key and a separate inverted lists are created for each term *t* in the object description. The inverted file for edge $e_{i}$ is the set of inverted lists containing objects lying on the edge, and there is an inverted file associated with every edge that contains at least one data object.

The proposed indexing framework has several features that significantly enhance GSTK query processing performance. First, the data objects located on edge $e_{id}$ are stored in inverted files and objects relevant to keyword queries can be easily retrieved using $\left(e_{id},t_{id}\right)$. Second, the distance between the starting node and data object is also stored in an inverted file, hence data objects can be accessed directly. Third, the set of fans for each data object $F_{D}$ is stored in the inverted file, enabling faster computation of the social relevance score. Fourth, upper-bound scores are computed considering the combined textual, social and spatial relevance to the query. Fifth, the binding component uses the upper-bound score for each edge to prune edges that do not contain data objects that could be in the top-*k* results.

### 4.3. Methodology

**Figure 5:**
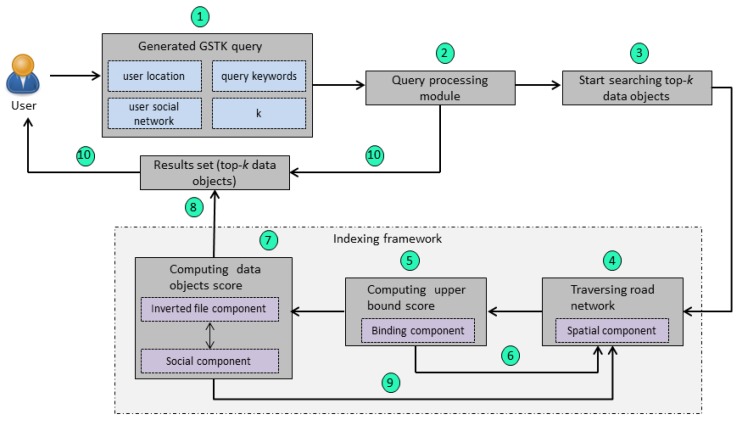
Overview of the proposed query processing methodology.

The overview of the proposed query processing methodology is shown in Figure 5. A user initially generates a GSTK query (Step 1 in Figure 5). The query processing module receives a query that has information about user location, query keywords, user social networks, and the number of requested data objects (*k*) (Step 2 in Figure 5). The query processing module then starts searching the top-*k* data objects (Step 3 in Figure 5); it utilizes the indexing framework to process the query. First, it accesses the spatial component to start traversing the road network and searches the candidate data objects from the edge where user is located (Step 4 in Figure 5). Next, the binding component is accessed to compute the upper-bound score of edge by using the pre-computed $\Omega_{t}$ and $\Omega_{f}$ (Step 5 in Figure 5). If the upper-bound score is less than the current *k*th data object, then the edge is pruned, and the proposed system continues traversing the adjacent edges (Step 6 in Figure 5). If the upper-bound score is greater than the current *k*th data object, then the query processing module accesses the inverted file and social components to compute the score of each data object that lies on the edge (Step 7 in Figure 5). Next, the result set is updated by inserting the data objects with a score greater than the current *k*th data object (Step 8 in Figure 5). The query processing module continues road network expansion to retrieve more candidate data objects that can be in the top-*k* result set (Step 9 in Figure 5). Finally, the system is terminated when all edges are explored or there is no edge left with an upper-bound score greater than the *k*th data object, and the result set is returned to the user (Step 10 in Figure 5).

### 4.4. Query Processing Algorithm

Algorithm 1 presents the main steps for the proposed Geo-Social Top-*k* Keyword query processing algorithm, GSTK-A. The algorithm takes input query $Q_{G}$ and returns result set $R_{k}$ containing the best *k* data objects in descending order by score ψ(d). GSTK-A expands adjacent edges of query objects in increasing order of distance from q.l similar to Dijkstra’s algorithm [41]. A min-heap *H* is implemented to arrange encountered edges which is initially empty. Each entry in *H* is represented as $\left(p_{r},e_{id}\right)$ where $p_{r}$ corresponds to the reference point in edge $e_{id}$ i.e., the starting point of expansion. An edge that contains query object *q* is represented as active edge $e_{active}$. Query object *q* becomes the reference point for an active edge and either adjacent node $n_{s}$ or $n_{e}$ becomes the reference point (or reference node) for other edges. Variable $m_{k}$ stores the score of the current *k*th data object in $R_{k}$.

The algorithm is initiated by visiting the active edge where query object *q* lies. Then, the $candsearch(\left(e_{id},t_{id}\right),m_{k})$ function finds candidate data objects $R_{c}$ lying on $e_{active}$ with $\psi \left(d\right)>m_{k}$, and updates $R_{k}$ and $m_{k}$ using the data objects in $R_{c}$. The traversal of adjacent edges continues until the min-heap *H* is exhausted or the shortest distance to any remaining data object produces an upper-bound score smaller or equal to $m_{k}$. The upper-bound score of a node *n* is computed using dist(n,q), the maximum textual and social relevance (which can be 1). Therefore, if the upper-bound score $\leq m_{k}$, then even if there is an unexplored data object *d* with maximum textual and social relevance, its score cannot be higher than the *k*th data object *d* in $R_{k}$ because dist(d,q.l)≥dist(n,q.l) since the algorithm strictly expands and selects the reference node with minimum distance to the query location.

Algorithm 2 presents the $candsearch(\left(e_{id},t_{id}\right),m_{k})$ procedure to identify candidate data objects. First, the algorithm accesses the social component to retrieve the friends of *q* and compute the social relevance score. Then, it computes the upper-bound score of the edges using the binding component with $\Omega_{t}$, $\Omega_{f}$, and the minimum distance from the query location to the edge. The inverted lists of term *t* are fetched only if their upper-bound scores are greater than $m_{k}$. The scores of data objects are computed using the formula in Equation (1), and data objects with $\psi \left(d\right)>m_{k}$ are returned.

Next, we discuss the running example of the proposed algorithm presented in Figure 1. For simplicity, we assume the example settings as discussed in Section 4.1, i.e., we have a set of users $U=\{u_{1},u_{2},\ldots ,u_{100}\}$. User $u_{1}$ is the query user *q* who issued a query with keywords “French restaurant”, requested one result (k=1), and has a set of ten friends $F_{q}=\left\{u_{4},u_{16},u_{23},u_{39},u_{48},u_{55},u_{67},u_{71},u_{80},u_{94}\right\}$. Equal preference is assigned to every attribute, i.e., α=β=γ=1 and δ=0.5. Recall that the textual relevance is the number of occurrences of the query words in the object description divided by the total number of keywords in the object description. Table 2 presents the details and score computation of $d_{2}$, $d_{3}$ and $d_{6}$.


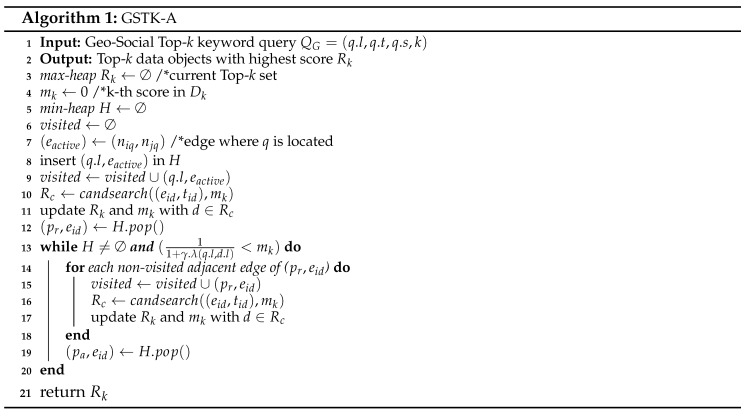



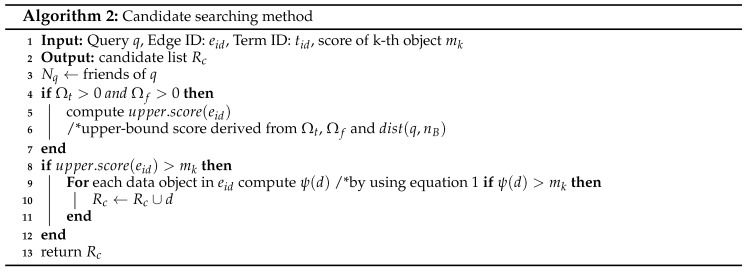


The algorithm starts network expansion from active edge $\left(n_{3},n_{8}\right)$ with *q* as the reference point. Edges $\left(q,n_{3}\right)$ and $\left(q,n_{8}\right)$ are inserted in min-heap *H*. First, $\left(q,n_{3}\right)$ and $\left(q,n_{8}\right)$ are explored and no data object is found in both edges. Then, $n_{3}$ becomes the reference point and edges $\left(n_{3},n_{2}\right)$, $\left(n_{3},n_{4}\right)$, and $\left(n_{3},n_{7}\right)$ are inserted in min-heap *H*. Next, the candsearch function searches the candidate data objects on edges $\left(n_{3},n_{2}\right)$, $\left(n_{3},n_{4}\right)$, and $\left(n_{3},n_{7}\right)$ having scores better than $m_{k}$. The upper-bound score of edge $\left(n_{3},n_{7}\right)$ is $\frac{0.5\times 0.61}{2}=0.15$ ($\Omega_{t}=0.5$, $\Omega_{f}=0.5\times \frac{23}{100}+0.5\times 1=0.61$ and $dist(q,n_{3})=2$). Therefore, the inverted list of edge is accessed because the upper-bound score of edge $\left(n_{3},n_{7}\right)$ is greater than the current $m_{k}$ score ($m_{k}=0$); moreover $d_{3}$ is retrieved with $\psi \left(d_{3}\right)=\frac{0.5\times 0.165}{4}=0.02$. Data object $d_{3}$ is inserted in the $R_{k}$ set, and $m_{k}$ is set to 0.02. The upper-bound score of edges $\left(n_{3},n_{2}\right)$ and $\left(n_{3},n_{4}\right)$ is zero, since there is only one data object $d_{4}$ found on $\left(n_{3},n_{4}\right)$ with a description (“cafe and bar”) that does not match with the query keywords. The algorithm continues expanding the edges whose upper-bound score is greater than $m_{k}$. Next, $n_{8}$ becomes the reference point and the edges $\left(n_{8},n_{1}\right)$, and $\left(n_{8},n_{9}\right)$ are explored, but neither edge contains objects relevant to the query keywords. Node $n_{2}$ becomes the next reference point and edges $\left(n_{2},n_{1}\right)$, and $\left(n_{2},n_{6}\right)$ are visited. There is no data object in $\left(n_{2},n_{1}\right)$ but the inverted list of $\left(n_{2},n_{6}\right)$ is accessed by candsearch function because the upper-bound score of $\left(n_{2},n_{6}\right)$ is $\frac{0.66\times 0.6}{4}=0.9$ which is greater than the current $m_{k}=0.02$. Therefore, any available candidate object could be the top-1 result. The inverted list of $\left(n_{2},n_{11}\right)$ is accessed and $d_{2}$ is retrieved with $\psi \left(d_{2}\right)=\frac{0.66\times 0.35}{7}=0.033$ which is greater than the current $m_{k}=0.02$. $R_{k}$ is updated with $d_{2}$ and set $m_{k}=0.033$. Next, $n_{4}$ becomes the reference point and edges $\left(n_{4},n_{5}\right)$, and $\left(n_{4},n_{10}\right)$ are explored. Edge $\left(n_{4},n_{5}\right)$ does not contain any data object, but the inverted list of $\left(n_{4},n_{10}\right)$ is accessed by candsearch function because upper-bound score of $\left(n_{4},n_{10}\right)$ is $\frac{1\times 0.545}{4}=0.136$ which greater than $m_{k}=0.033$. Data object $d_{6}$ is found with $\psi \left(d_{6}\right)=\frac{1\times 0.145}{6}=0.024$, which is less than $s_{k}=0.033$. Hence $R_{k}$ and $m_{k}$ are not updated. The algorithm continues expanding the network until min-heap *H* is exhausted or the minimum network distance to any remaining data object produces an upper-bound score smaller or equal to $m_{k}$. The upper-bound score of remaining edges is 0 because they do not contain any data objects relevant to the query keywords. Consequently, the algorithm terminates and reports $d_{2}$ as the top-1 result.

### 4.5. Index Maintenance

In general, the updates in the textual description and check-in information of data objects are more recurrent than the updates in the spatial information of data objects or road networks. Therefore, we first discuss index maintenance when the textual description of data object is modified. Without loss of generality, let us assume that given a data object d∈D with textual description d.t is to be modified with new textual description $d.t^{+}$. The update procedure begins by finding the edge $e_{id}$ in which *d* is located using the network’s R-tree. Then by using $e_{id}$, the vector is obtained that contains all terms $e_{id}.t$ in that edge. Next, for terms $t_{i}$ that are in d.t but not in $d.t^{+}$ data object *d* is removed from the inverted list of $t_{i}$. The significance of term $t_{i}$ in $e_{id}.t$ is recomputed using the inverted list of $t_{i}$, when $\Omega_{t_{i},e_{id}}=\Omega_{t_{i},d}$ and *d* is deleted from the inverted list of $t_{i}$. For terms $t_{j}$ that are in $d.t^{+}$ but not in d.t, *d* is inserted into the inverted list of $t_{j}$. The significance of term $t_{j}$ in $e_{id}.t$ is recomputed using the inverted list of $t_{j}$, when $\Omega_{t_{j},d}>\Omega_{t_{i},e_{id}}$, and *d* is inserted into the inverted list of $t_{j}$. For terms $t_{k}$ that are in both d.t and $d.t^{+}$, the significance of $t_{k}$ ($\Omega_{t_{k},d}$) in the inverted list is updated to ($\Omega_{t_{k}^{+},d}$). The significance of term $t_{k}$ in $e_{id}.t$ is also updated to a significance of ($\Omega_{t_{k}^{+},d}$) when $\Omega_{t_{k}^{+},d}>\Omega_{t_{k},d}$.

Now we discuss index maintenance for an update in the check-in information of data object *d*. Consider a user $u_{i}$ checked-in to data object $d_{i}\in D$. Similarly, the update procedure starts with retrieving edge $e_{id}$ where $d_{i}$ lies and then vector $e_{id}.t$ is accessed. Next, the inverted lists of all terms are accessed that are in $d_{i}.t$ using $\left(e_{id},t_{id}\right)$. If $u_{i}$ is already a fan of $d_{i}$ i.e., $u_{i}\in F_{d_{i}}$, no further action is required. If user $u_{i}\notin F_{d_{i}}$, it is inserted in $F_{d_{i}}$. Next, the highest significance of fans $\Omega_{f}$ on $e_{id}$ with terms $d_{i}.t$ is updated if $\Omega_{f_{i}}>\Omega_{f}$ where $\Omega_{f_{i}}$ indicates the $\Omega_{f}$ of $d_{i}$ after inserting new fan $u_{i}$ in $F_{d_{i}}$. Next, we discuss the updates in the social relationships which are relatively straight forward. Consider users $u_{j}$ and $u_{k}$ becomes friends, $u_{j}$ is then added in $N_{u_{k}}$ and vice versa. Similarly, if they unfriend each other, $u_{j}$ is deleted from $N_{u_{k}}$ and vice versa. If $u_{j}$ deletes his or her account, then he or she is removed from $N_{u}$ of all their friends and also removed from fan lists of all such data objects *d* where $u_{j}\in F_{d}$.

Finally, we discuss the updates in the spatial information of the data object which is a relatively infrequent operation. Consider a data object $d_{added}$ with description $d_{added}.t$ located on edge $e_{id}$. As mentioned previously, initially edge $e_{id}$ is located and vector $e_{id}.t$ is accessed. Next for the term $t_{i}$ that is present in both $d_{added}.t$ and $e_{id}.t$, the inverted list of $t_{i}$ is accessed and data object $d_{added}$ is inserted along with its $dist(n_{s},d_{added})$, $\Omega_{t_{i},d_{added}}$, and $F_{d_{added}}$. Next, the significance of term $t_{i}$ in $e_{id}.t$ is updated if $\Omega_{t_{i},d_{added}}>\Omega_{t_{i},e_{id}}$. Then, the highest significance of fans $\Omega_{f}$ on $e_{id}$ with term $t_{i}$ is updated if $\Omega_{f_{added}}>\Omega_{f}$ where $\Omega_{f_{added}}$ indicates the $\Omega_{f}$ of $d_{added}$. For term $t_{j}$ that are in $d_{added}.t$ but not in $e_{id}.t$, a new inverted list ($e_{id},t_{j}$) is created, and a data object $d_{added}$ is inserted into it. Next, $\Omega_{t}$ and $\Omega_{f}$ are computed for term $t_{j}$ on edge $e_{id}$. Assume data object $d_{deleted}$ with $d_{deleted}.t$ to be removed is located on $e_{id}$. All inverted lists with $d_{deleted}.t$ are accessed and $d_{deleted}$ is removed from them. Next, the significance of term $t\in d_{deleted}.t$ in $e_{id}.t$ is updated if $\Omega_{t,d_{deleted}}>\Omega_{t,e_{id}}$, and $\Omega_{f}$ on $e_{id}$ with term *t* is updated if $\Omega_{f_{deleted}}>\Omega_{f}$ where $\Omega_{f_{deleted}}$ indicates the $\Omega_{f}$ of $d_{deleted}$. Finally, updating spatial location of a data object *d* is handled as a deletion followed by an insertion.

## 5. Geo-Social Skyline Keyword Queries

### 5.1. Problem Formulation

Skyline queries are useful for extracting desired data objects from a multi-dimensional datasets. A data object is desired if it is not dominated by any other data object i.e., it is not worse than any other data object in all dimensions. Geo-Social Top-*k* keywords queries retrieve the *k* best data objects based on spatial, textual and social relevance to query *q*. GSTK uses a scoring function and results depends on the values of query parameter (α, β, γ) defined by the user. However, it is important that the user has adequate knowledge to select appropriate values for these parameters. It is somewhat challenging to define a suitable scoring function due to different attribute distributions or user inability to choose an appropriate values [42]. Therefore, to supplement GSTK queries, we extend our work to study Geo-Social Skyline Keyword (GSSK) queries. GSSK queries return every object *d* within range *r* (i.e., dist(q,d)<r) which is not dominated by any other object in terms of distance to the query location and aggregated score of social and keyword relevance. The range parameter is used in GSSK queries to accommodate the case where a user is not interested in distant places but wants to find all possible objects within the chosen range.

The aggregated social and keyword and keyword score σ(d) is defined as:

(3) σ(d)=μ(d.t,q.t)×γ(d.s,q.s)

where μ(q.t,d.t) is the textual similarity between q.t and d.t and τ(q.s,d.s) is the social relevance of data object *d* with respect to query *q*. The definition of μ(q.t,d.t) and τ(q.s,d.s) are presented in Section 4.1. To calculate τ(q.s,d.s) we set δ=0.5 to indicate equal importance to query user friends and other users.

**Geo-Social Keyword Dominance:** A data object *d* is dominated by another data object $d^{{}^{\prime }}$ if $\sigma \left(d^{{}^{\prime }}\right)\geq \sigma \left(d\right)$, $dist\left(q,d^{{}^{\prime }}\right)\leq dist\left(q,d\right)$ and at least one of the following holds: $\sigma \left(d^{{}^{\prime }}\right)>\sigma \left(d\right)$ and $dist\left(q,d^{{}^{\prime }}\right)<dist\left(q,d\right)$. We denote the dominance relationship as $d^{{}^{\prime }}≺d$ which implies that data object *d* is dominated by data object $d^{{}^{\prime }}$.

Geo-Social Skyline Keyword Queries: Given a query *q*, a set of keywords q.t and range *r*, GSSK queries return all data objects that are not dominated by any other data object.

**Mapping to Distance-Score Space:** Each data object *d* is mapped to a point in the distance-score space denoted by *M*, defined by axes dist(q,d) and σ(d).

**Figure 6:**
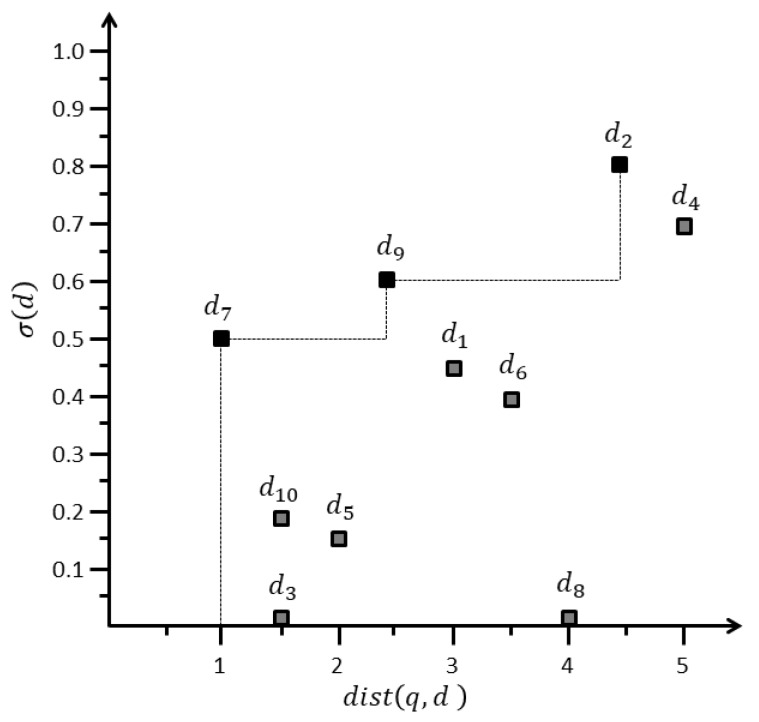
Distance-score mapping.

To describe the problem definition, consider we have a set of data objects $d=\{d_{1},d_{2},\ldots ,d_{10}\}$ inside range r=5, with dist(q,d) and σ(d), as presented in Table 3. Figure 6 illustrates the mapping of data objects presented in Table 3 to the distance-score space *M*. Horizontal and vertical axes represent distance dist(q,d) and score σ(d), respectively. Lower values are preferred for the distance, whereas higher values are preferred for the score. Figure 6 illustrates that $d_{2}$, $d_{7}$, and $d_{9}$ are dominant data objects, and all other data objects are dominated by them. For example, $d_{2}$ dominates $d_{4}$ because $\sigma \left(d_{2}\right)>\sigma \left(d_{4}\right)$ and $dist\left(q,d_{2}\right)<dist\left(q,d_{4}\right)$. Similarly, $d_{7}$ dominates $d_{5}$ because $d_{7}$ has a superior distance and score. Therefore, $d_{2}$, $d_{7}$, and $d_{9}$ are skyline objects $s_{i}$, belonging to skyline set *SKY* i.e., $\{d_{2},d_{7},d_{9}\}\in SKY$.

### 5.2. Query Processing Algorithm

We use the same indexing framework as described in Section 4.2, except the upper-bound score of the edge is composed from $\Omega_{t}$ and $\Omega_{f}$. The inverted list of a term *t* on edge $e_{id}$ is only accessed if the edge is not dominated by a data object $s_{i}\in SKY$. An edge is dominated by $s_{i}$ if the upper-bound score of the edge is less than $\sigma (s_{i})$ and $dist(q,n_{B})$ is greater than or equal to $dist(q,s_{i})$. We then use Lemma 1 to prune non-skyline objects. In addition, the query processing methodology of GSSK query is similar to GSTK query as presented in Section 4.3.

**Lemma** **1.** *If edge $e_{id}$ is dominated by $s_{i}\in SKY$, then edge $e_{id}$ does not contain any skyline objects.*

**Proof.**  Data object $s_{i}$ represents the object in the skyline set, so if edge $e_{id}$ is dominated by $s_{i}$ then the upper-bound score of edge $e_{id}$ is less than the score of $s_{i}$ and $dist\left(q,n_{B}\right)>dist\left(q,s_{i}\right)$. Thus, all objects that lie on the edge have scores lower than the upper-bound score, and hence they cannot be skyline objects. □

We now present an algorithm *GSSK-A* for processing Geo-Social Skyline Keyword queries that returns the set of skyline objects SKY within range *r*. Algorithm 3 is similar to Algorithm 1; it traverses the road network in a similar fashion and exploration begins from the active edge $e_{active}$ where *q* is located. The same min-heap *H* is implemented to arrange the encountered edges and it is initially empty. For each unexplored adjacent edge whose minimum distance is less than range *r* (i.e., $dist(q,n_{B})\leq r$), we compute the upper-bound score based on $\Omega_{t}$ and $\Omega_{f}$. The inverted list of a term *t* on edge $e_{id}$ is only accessed if the edge is not dominated by any $s_{i}\in SKY$. Next, for each data object *d* that lies on edge $e_{id}$, σ(d) is computed if it satisfies the range constraint *r* (i.e., dist(q,d)≤r). If data object *d* is not dominated by any skyline object $s_{i}\in SKY$, it is added to skyline set SKY. Finally, the algorithm terminates when the heap is exhausted or there is no edge whose minimum distance is within range *r*.


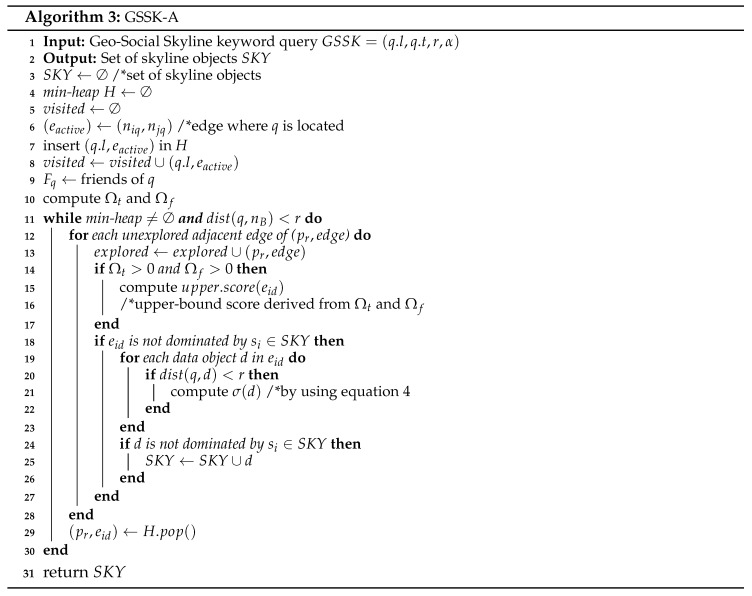


## 6. Performance Evaluation

In this section, we evaluated the performance of our proposed algorithm through simulation experiments. Section 6.1 describes the experiment settings, and Section 6.2 and Section 6.3 present the experimental results for GSTK and GSSK queries, respectively. Section 6.4 compares GSTK and GSSK query responses.

### 6.1. Experimental Settings

A real road network dataset [43] was used that comprised the main roads of North America, with 175,812 nodes and 179,178 edges. The real dataset of Gowalla [44] and a synthetic dataset were used in these experiments. The characteristics of these datasets are presented in Table 4. Gowalla was a geo-social networking website that was subsequently acquired by Facebook. It included 196,591 users, 950,327 friendships, 6,442,890 check-ins and 1,280,956 checked-in places (data objects). We randomly generated data objects on each edge, producing seven data objects on average for each edge. We extracted data object descriptions from Twitter messages [45], and one tweet per data object was assigned. A user who checked-in at the location was considered a fan. For each experiment, we randomly selected 100 query objects from users and report the average cost of 100 queries. Query keywords were random terms generated from the dataset vocabulary. Table 5 summarizes the experiment parameters. In each experiment, we varied a single parameter within the given range, holding all other parameters at the constant default value highlighted in bold text unless specified otherwise.

To the best of our knowledge, the processing of GSTK and GSSK queries has not been studied before for road networks. Therefore, we compared our algorithms with social-textual index (SNIR-tree) proposed by Wu et al. [10] which was applicable for Euclidean space. For a fair comparison, we modified their work and designed a competitive technique (INE-SNIR) comprising the road network framework (INE) proposed by Papadia et al. [40] and social-textual index (SNIR-tree) [10]. The road network framework [40] enables locating a query point and traversing the network. The SNIR-tree [10] is based on the IR-tree [5] and is used to index and store user information and relationships; it also stores the social, textual and spatial information of data objects. The SNIR-tree index also facilitates finding the data objects inside the minimum bounding region (MBR) of edges socially and textually relevant to the query. The query processing of INE-SNIR works as follows. The MBR of edge $MBR(e_{id})$ is used to perform a query in the index to obtain the data objects inside $MBR(e_{id})$ that are socially and textually relevant to the query, and then utilizes the spatial framework to calculate the distance between the query location and data object.

We implemented all algorithms in Java, and experiments were conducted on a PC machine with a 3.60-GHz Intel Core i7 process and 16 GB RAM. Indexes were disk-resident and the page size of the network’s R-Tree and SNIR-tree were 8KB. We evaluated the algorithms performance using the following measures: (1) runtime, which indicates the total query execution time and (2) I/O cost, which represents the number of disk page accessed for query processing.

### 6.2. Experimental Results of Geo-Social Top-k Keyword Queries

**Figure 7:**
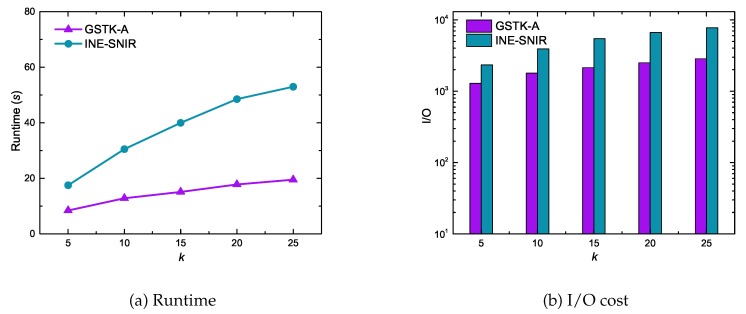
Effect of *k* on runtime and I/O cost.

Figure 7 shows the performance of GSTK-A and INE-SNIR with respect to *k*, the number of requested data objects with the highest scores. Experimental results reveal that the runtime and I/O cost of both the algorithms increased with increasing *k*, which is unsurprising since more data objects are explored and processed when increasing *k*. However, GSTK-A significantly outperformed INE-SNIR due to the proposed indexing framework. First, fewer inverted lists were processed due to the upper-bound score and, second, score calculation was highly efficient because the inverted list stores $dist(n_{s},d_{i})$, $\Omega_{t_{i},d_{i}}$ and $F_{d_{i}}$.

**Figure 8:**
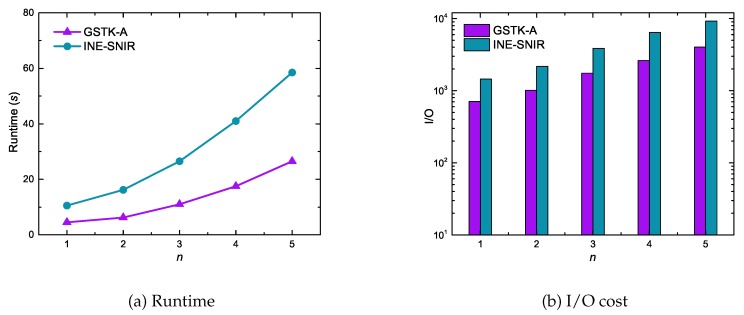
Effect of the number of keywords on runtime and I/O cost.

Figure 8 depicts the runtime and I/O cost performance for GSTK-A and INE-SNIR with respect to the number of query keywords. As expected, the runtime and I/O cost for both algorithms increased with an increasing number of keywords in the query. With fewer query keywords, fewer data objects are relevant to the query, hence fewer edges and data objects are explored and processed. In contrast, with more query keywords, more data objects are relevant and consequently more edges and data objects are explored and verified. Notice that runtime and I/O cost for INE-SNIR increased more rapidly than for GSTK-A because the INE-SNIR candidate searching process is quite expensive. First it requires searching an index to retrieve data objects inside the MBR of edge that are textually and socially relevant to the query, and then computes the network distance between the query and data objects.

**Figure 9:**
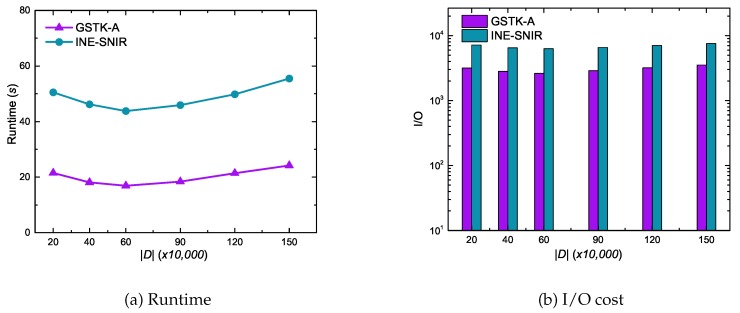
Effect of the number of data objects on runtime and I/O cost.

We randomly generated 200 K, 400 K, 600 K, 900 K, 1200 K, and 1500 K data objects on the synthetic dataset to produce various sized datasets to evaluate the scalability of GSTK-A and INE-SNIR algorithms, as depicted in Figure 9. Both algorithms exhibited relatively poor performance when the number of data objects was small or large. Performance degraded for small number of data objects mainly because data object density is low and the algorithms expand more edges to retrieve *k* data objects. On the other hand, when the number of data objects is large, data objects relevant to query keywords also increase, with consequential increases runtime and I/O cost. The algorithms performed best for 400–600 K data objects.

**Figure 10:**
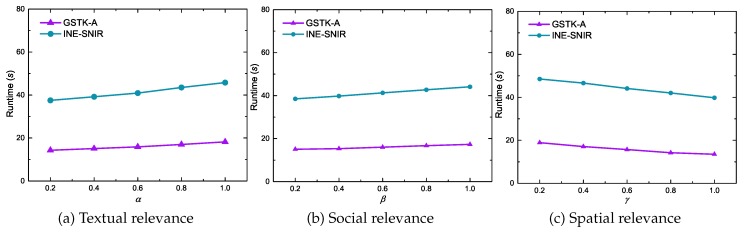
Effect of query parameters on runtime.

Figure 10 illustrates the impacts from query preference parameters α,β and γ on GSTK-A and INE- SNIR runtime. For each case, we varied the value of one parameter while maintaining the other two parameters at 0.5. Query preference parameters do not have significant impact on running time, although the performance of both approaches slightly improve when γ (spatial relevance) has a high preference, because fewer edges are required to be expanded; the performance slightly degrades when increasing α and β (textual and social relevance, respectively) due to the large number of data objects relevant to the query.

### 6.3. Experimental Results of Geo-Social Skyline Keyword Queries

In this section we analyzed the performance of the algorithms (*GSSK-A*) for geo-social skyline keyword queries. We implemented the SKY-SNIR algorithm which uses the same indexing structure composed of INE [40] and SNIR-tree [10]. The SKY-SNIR algorithm first uses an SNIR-tree index to compute the aggregated score of data objects within range *r* based on social and textual relevance, and then calculates the network distance between the query and data objects. Skyline objects are then identified as those objects that are not dominated by any other data object using the aggregated score and network distance.

**Figure 11:**
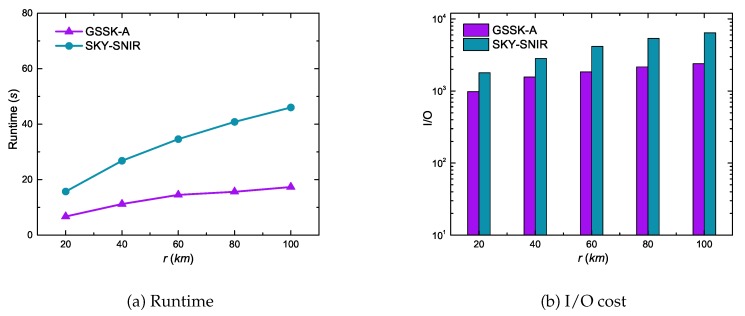
Effect of range on runtime and I/O cost.

Figure 11 illustrates the range effects on GSSK-A and SKY-SNIR performance. Runtime and I/O cost increased with increasing *r* for both algorithms because the search space increased as *r* increasesd; consequently, the algorithms needed to expand more edges and process more data objects. However, GSSK-A scaled much better than SKY-SNIR because it only expanded edges that were not dominated by skyline data objects.

**Figure 12:**
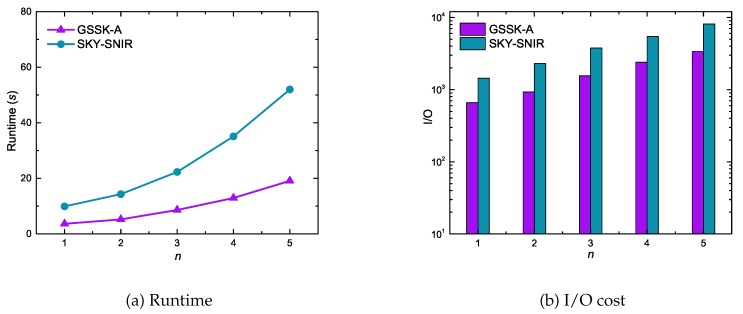
Effect of the number of keywords on runtime and I/O cost.

Figure 12 compares GSSK-A and SKY-SNIR performance with respect to the number of query keywords. These experiment results indicate similar trends as in Figure 8. The proposed algorithm GSSK-A not only outperformed SKY-SNIR but also scaled more effectively because SKY-SNIR is expensive for searching an index to retrieve data objects.

**Figure 13:**
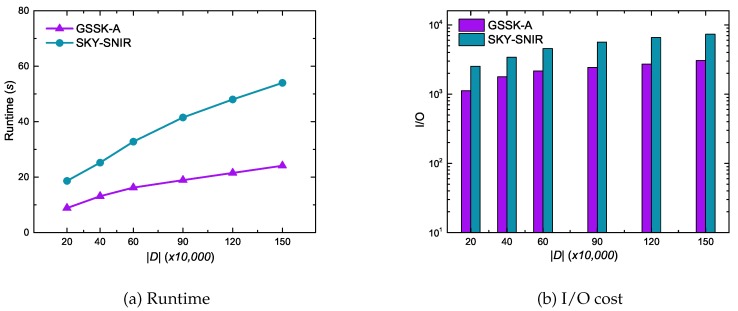
Effect of the number of data objects on runtime and I/O cost.

Figure 13 shows data object cardinality effects on GSSK-A and SKY-SNIR performance using the synthetic dataset. In contrast to the experimental results of the GSTK query (Figure 9), the results of the GSSK query reveals that the runtime and I/O cost gradually increased as the cardinality of data objects increased. This is primarily because GSTK queries retrieve the *k* best data objects, and the algorithms must expand more edges when the search space is less dense, which increases runtime and I/O cost. In contrast, GSSK queries retrieve all data objects in *r* that are not dominated by any other data object, hence more data objects must be explored and verified as cardinality increases.

### 6.4. Comparison of Results Returned by GSTK and GSSK Queries

This section compares and analyzes the results returned by GSTK and GSSK queries. The main advantage of the GSTK query is that user can control the number of data objects to be returned. However, the user must able to define the scoring function correctly, which can be challenging. GSSK queries solve that problem and do not need a scoring function, and also only provide data objects within the user specified range. However, the number of data objects in a result set cannot be controlled by the user. In the next experiment, we executed 100 queries for each setting, reporting the average number of data objects retrieved by GSTK, GSSK and the average number of common data objects that were retrieved by both queries.

**Figure 14:**
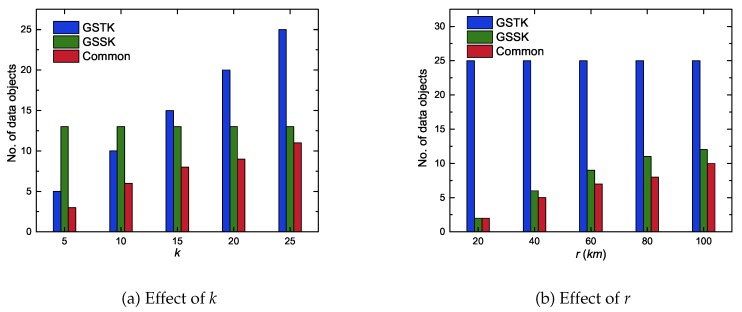
The number of data objects returned by GSTK and GSSK queries.

Figure 14a, illustrates the GSTK and GSSK result set outcomes with respect to *k* for r=100. It is obvious that GSSK returned the same number of skyline data objects within range regardless of *k*, whereas GSTK returned exactly *k* data objects. Figure 14b, depicts the GSTK and GSSK result set outcomes with respect to *r* for k=25. As with fixed *r*, GSTK always returned the same *k* number of data objects, whereas the number of data objects in the result set of GSSK increased as *r* increased. The experimental results demonstrate that the result set of GSTK and GSSK shared many data objects but also included data objects that the other query failed to return. Thus, the proposed GSSK and GSTK queries complemented each other.

## 7. Conclusions

This paper introduced geo-social top-*k* keyword (GSTK) queries for road networks for the first time, integrating social relevance into traditional spatial keyword search and returning the *k* best data objects based on spatial, textual, and social relevance to the query. We also extended the model to propose geo-social skyline keyword queries (GSSK) on road networks, which returns all data objects that are not dominated by any other data object. We developed an efficient indexing structure that effectively prunes the search space by retrieving data objects relevant to the query, and proposed efficient algorithms to process GSTK and GSSK queries in road networks. Experimental results demonstrates that our proposed approaches significantly outperforms INE-SNIR and SKY-INIR algorithms in terms of query runtime and I/O cost.

The proposed study retrieves data objects based on the single search keywords such as “French restaurant.” In the future, we plan to extend our work to study geo-social top-*k* collective keyword queries, which will retrieve a group of *k* data objects based on the set of keywords, query location, and query social information. Geo-social top-*k* collective keyword query has many real-world applications as the user often needs to find a group of data objects such as “tourist attractions,” “shopping malls,” and “cafes.” The processing of geo-social top-*k* collective keyword query is more challenging as it has to consider all sets of query keywords, data objects in the result set should be close to the query location, and should have minimum inter-object distance.

## Figures and Tables

**Table 1:** Frequently used notations.

Notation	Definition
ψ(d)	Score of data object *d*
μ(q.t,d.t)	Textual relevance of data object *d* with query keywords
τ(q.s,d.s)	Social relevance of data object *d* with query user
λ(q.l,d.l)	Spatial relevance of data object *d* with query location
(α,β,γ)	Preference parameters that represent the importance of textual, social and spatial relevance, respectively
δ	Preference parameter that controls the importance between query user friends and other users
$F_{d}$	Set of fans of data object *d*
$N_{q}$	One-hop neighbors of *q* in social network
$\Omega_{t}$	Highest significance of a given term *t* among the description of the data objects lying on edge $e_{id}$
$\Omega_{f}$	Highest significance of fans of any data object lying on edge $e_{id}$ with term *t*
σ(d)	Aggregated social and textual score used in GSSK queries

**Table 2:** Score computation of data objects.

*d*	λ(q.l,d.l)	μ(q.t,d.t)	$F_{D}$	*$N_{q}$* in *$F_{D}$*	τ(q.s,d.s)	ψ(d)
$d_{2}$	7	0.66	20	5	0.35	0.033
$d_{3}$	4	0.50	23	1	0.165	0.020
$d_{6}$	6	1	9	2	0.145	0.024

**Table 3:** Data object details.

Data Object	$\mathrm{dist}(q,d_{i})$	Cscore
$d_{1}$	3	0.45
$d_{2}$	4.5	0.8
$d_{3}$	1.5	0
$d_{4}$	5	0.7
$d_{5}$	2	0.15
$d_{6}$	3.5	0.4
$d_{7}$	1	0.5
$d_{8}$	4	0
$d_{9}$	2.5	0.6
$d_{10}$	1.5	0.2

**Table 4:** Summary of dataset.

Attribute	Gowalla	Synthetic
Total Size	1.62 GB	1.87 GB
Total data objects	1,280,956	1,500,000
Total users	196,591	150,654
Total friendships	950,327	830,683
Total check-ins	6,442,890	7,364,130
Average fans per data object	3.4	5.8
Total words	8,198,118	11,420,957
Total distinct words	798,118	112,957
Average distinct words per data object	4.8	6.2

**Table 5:** Experimental parameter settings.

Parameter	Values
Number of results (*k*)	5, 10, **15**, 20, 25
Number of keywords (*n*)	1, 2, **3**, 4, 5
Number of data objects (|D|)	20, 40, 60, 90, 120, **1300**, 1500 (x10,000)
Range (*r*)	20, 40, **60**, 80, 100
α,β,γ	0.2, 0.4, 0.6, 0.8, **1.0**
δ	0.5
